# First-Line Gemcitabine Plus Cisplatin in Nonsmall Cell Lung Cancer Patients

**DOI:** 10.1155/2014/960458

**Published:** 2014-01-23

**Authors:** Ying Li, Lin Run Wang, Jian Chen, Yan Lou, Guo Bing Zhang

**Affiliations:** The First Affiliated Hospital, College of Medicine, Zhejiang University, Hangzhou 310003, China

## Abstract

*Aim*. To evaluate the predictive value of RRM1, ERCCl, and BRCA1 expression in Chinese NSCLC patients treated with gemcitabine and cisplatin. *Methods*. Real-time fluorescent quantitative PCR was used to determine the RRM1, ERCC1, and BRCA1 mRNA expression levels of peripheral blood in late-stage NSCLC patients. The relationship between peripheral blood and mRNA expression in tumor tissues was analyzed further. *Results*. In terms of the tumor susceptibility to chemotherapy, the response rate in the low-RRM1-expression group was significantly greater than in the high-expression group (52.9% versus 5.9%, *χ* ^2^ test, *P* = 0.007). Subjects with low peripheral blood RRM1 expression survived longer than those with high RRM1 expression (15.5 versus 12.0 months, logrank 3.980, *P* = 0.046). Linear correlations were observed between peripheral blood and tumor tissue expression levels for RRM1 (*R* ^2^ = 0.045, *P* = 0.048) and BRCA1 (*R* ^2^ = 0.021, *P* = 0.001). *Conclusion*. Our study demonstrates increased survival and superior efficacy of gemcitabine and cisplatin combination chemotherapy in the treatment of NSCLC patients with low peripheral blood RRM1 expression. The linear correlations of the relative expression of mRNA were observed between peripheral blood and tumor tissue expression levels for RRM1 and BRCA1. RRM1 gene expression may contribute to chemotherapy sensitivity and may be an indicator of survival. It was significant to individual chemotherapy of patients with advanced NSCLC who do not have sufficient tumor tissue.

## 1. Introduction

In recent years, research has focused on abnormal DNA repair and drug resistance for individualized treatment of lung cancer. Ribonucleotide reductase (RR) catalyzes the formation of deoxyribonucleotides from ribonucleotides. It plays a key role in repairing DNA and it is also an important rate-limiting enzyme during DNA synthesis. The M1 subunit of RR (RRM1) is the nucleotide-binding site, which controls substrate specificity and enzyme activity. RRM1 also is the binding site for chemotherapy drugs such as gemcitabine and other nucleoside analogues [1]. Preclinical studies have shown that patients with low tumor tissue RRM1 expression had greater median length of disease progression and median survival time than did patients with high tumor tissue RRM1 expression. RRM1 expression level is associated with gemcitabine resistance in tumor cells [2, 3]. Therefore, RRM1 expression level is an important benchmark for the selection of patients for gemcitabine chemotherapy.

The excision repair cross complementation 1 gene (ERCCl) is involved in DNA damage repair and excision caused by cisplatin drugs. In recent years, increasing attention was to the relationship between ERCC1 expression level and cisplatin-based chemotherapy sensitivity. A retrospective study showed that ERCC1 mRNA expression levels were negatively correlated with cisplatin drug efficacy, which indicated that ERCC1 mRNA levels could be used as an independent prognostic indicator. ERCC1 is a critical gene used for the evaluation of cisplatin resistance [4, 5].

The breast cancer susceptibility gene 1 (BRCA1) is involved in the process of mitosis and also plays a predominant role in the repair of DNA damage. Some studies demonstrated that NSCLC patients with high BRCA1 mRNA expression had low efficacy with cisplatin therapy but high efficacy with the antimicrotubule drugs docetaxel and vinorelbine, which suggested that BRCA1 may be an important prognostic indicator and predictive factor for chemotherapy success in NSCLC patients [6, 7].

However, as demonstrated from the multitude of clinical studies, quantitative analysis of RRM1, ERCC1, and BRCA1 mRNA expression levels has been performed mainly on tumor specimens obtained from fiberoptic bronchoscopy biopsy or surgical resection. However, for patients with cytologically confirmed diagnoses of advanced NSCLC but without sufficient amount of tumor tissue sample, an effective detection method has not yet been developed. In our previous study, we found the relationship between the expression of RRM1 and BRCA1 in peripheral blood and the sensitivity of platinum plus gemcitabine [8]. In order to further investigate the association between gene expression in peripheral blood and the efficacy of gemcitabine and carboplatin combination chemotherapy, we collected a series of peripheral blood samples and tumor tissue from Chinese advanced NSCLC patients treated with gemcitabine plus platinum. In addition, we evaluated relationships between tumor tissue and peripheral blood gene expression in an attempt to guide the selection of patients for chemotherapy. This would ultimately improve drug treatment efficiency and reduce unnecessary drug side effects and economic costs and improve individualized treatment.

## 2. Materials and Methods

### 2.1. Patients

We enrolled 40 advanced-stage IIIB and IV NSCLC cases who did not qualify for total surgical resection and instead received chemotherapy. Among them, three subjects dropped out of treatment due to drug side effects, and three subjects switched to other pharmacotherapies. Liver, kidney, and bone marrow function met the following requirements: blood transaminase <2 times normal, WBC >4.0 × 10^9^/L, platelets >100 × 10^9^/L, and serum creatinine <1.5 times normal. The physical status scores for all cases were ≤2 based on Zubrod-ECOG-WHO standards, and the expected survival was > three months. The cases included 25 males and 9 females, aged 40–80, with a median age of 61 years. And 13 cases were smokers while 21 cases were nonsmokers. The performance status (PS) was as follows: 9 cases got 0 point, 24 cases got 1 point, and 1 case got 2. And there were 10 cases of squamous cell carcinoma and 24 cases of adenocarcinoma. Nine were stage IIIB and 25 stage IV; 11 were locally advanced and 23 metastatic stage IIIB or IV NSCLC patients. Then, every patient received standard first-line chemotherapy: gemcitabine 1,200 mg/m^2^ (d1, d8) and carboplatin (AUC5; d1) every three weeks. Patient evaluations were carried out at baseline and after two cycles of chemotherapy. The primary endpoint of this study was overall response rate, which was defined as confirmed complete and partial responses according to RECIST criteria. The secondary endpoint was overall survival (OS). These were calculated from the start of therapy to the date of death in the case of OS. This study was approved by the Ethics Committee of the First Affiliated Hospital, School of Medicine, Zhejiang University. And all patients signed informed consent before enrollment in the study.

### 2.2. Sample Preparation

Prior to the first course of chemotherapy, 3 mL of peripheral blood was taken in EDTA anticoagulant. After centrifugation, the cell layer was washed twice in erythrocyte lysis buffer followed by a PBS wash. The precipitated cells were then treated with 600 *μ*L Trizol, mixed with the chloroform. After centrifugation, the upper layer was transferred to another tube, mixed with isopropanol, and then centrifuged once more. Subsequently, the precipitate was dried in vacuum after washing with 75% ethanol, and then dissolved in DEPC water. About 100 mg of tissue sample was frozen in liquid nitrogen and dissociated in a mortar. After Trizol treatment, total RNA was extracted as the blood samples. First-strand cDNA was synthesized from total RNA samples using Promega kits in accordance with the kit instructions. cDNA samples were stored at −20°C.

### 2.3. Primer Design and Synthesis

Primers were designed based on the GeneBank sequence of RRM1 (AF107045), ERCC1 (AF001925), BRCA1 (U14680), and the housekeeping gene *β*-actin (AY582799).

The RRM1 upstream primer was 5′-ACTAAGCACCCTGACTATGCTATCC-3′, downstream primer 5′-CTTCCATCACATCACTGAACACTTT-3′; ERCC1 upstream primer 5′-CTGGGAATTTGGCGACGTAA-3′, downstream primer 5′-ATGGATGTAGTCTGGGTGCAG-3′; BRCA1 upstream primer 5′-CCCATTTTCCTCCCGCA-3′, downstream primer 5′-GGACCTTGGTGGTTTCT-3′; *β*-actin housekeeping genes upstream primer 5′-TGAGCGCGGCTACAGCTT-3′, downstream primer 5′-TCCTTAATGTCACGCACGATTT-3′. The primers were synthesized by Shanghai Sangon Company.

### 2.4. Real-Time Quantitative PCR

Using the qPCR kit (Takara, Beijing, China), the standard plasmid RRM1, ERCC1, BRCA1, and *β*-actin were diluted into six different concentrations followed by fluorescence real-time quantitative PCR. The following reagents were added to the reaction mixture: 22.5 *μ*L of Real Master Mix/SYBR Solution, 1 *μ*L each of the upstream and downstream primers, 0.5 *μ*L of Rox reference dye, 5 *μ*L of template DNA, and 20.5 *μ*L of DEPC water. Reaction conditions were as follows: polymerase activation for 20 seconds at 95°C and 40 cycles of PCR amplification for 5 seconds at 95°C or 31 seconds at 60°C.

### 2.5. Statistical Methods

The SPSS 15.0 statistical software was used for data analysis. During statistical analysis of RRM1, ERCC1, and BRCA1, the expression levels (relative to *β*-actin) were divided into the high- and low-expression groups where the boundary was defined by the median. The *χ* ^2^ test was performed for categorical variables and Spearman's rank correlation analysis was performed for continuous variables. Survival analysis was performed using Kaplan-Meier survival curves and logrank test methods. Factors such as age, sex, smoking, type of organization, and gene expression level were analyzed by independent predictors. After logrank test methods univariate analysis, factors with significant differences were analyzed by cox-regression methods for multivariate analysis. *P* < 0.05 defined statistically significant differences.

## 3. Results

### 3.1. Patient Characteristics and Clinical Outcome

**Figure 1:**
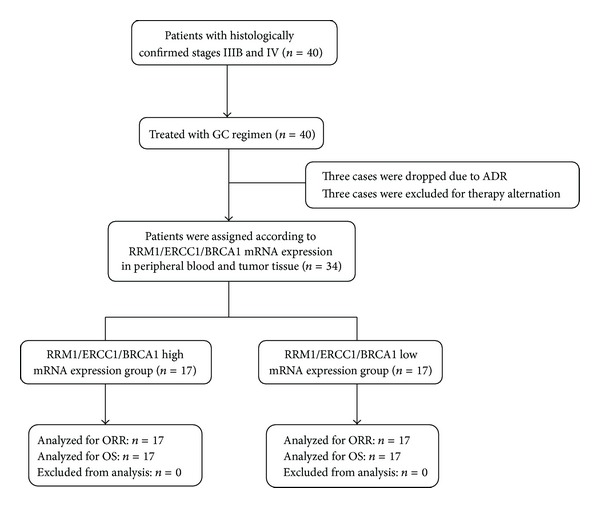
Consort diagram: GC regimen: gemcitabine 1,200 mg/m^2^  (d1, d8) and carboplatin (AUC5; d1) every three weeks; ADR: adverse drug reaction.

Thirty-four patients were included, with median age 61 (40–80), 74% male, 74% stage IV, 29% stage IIIB, 38% smokers, 29% squamous cell carcinoma, 71% adenocarcinoma, 26% ECOG0, 71% ECOG1, and 3% ECOG2. Clinical data and samples from primary tumors and peripheral blood were available for 22 patients, who were included in the present study. Amplification of RRM1, ERCC1, and BRCA1 was successful in 34 samples of advanced-stage NSCLC. Patient characteristics are shown in Table 1. The outcomes for the 34 patients assessed in the present study include the following: 10 partial responses (29.4%), 15 stable disease (44.1%), and 9 progression disease (26.5%). The overall objective response rate was defined as confirmed complete and partial responses. The response rate was 29.4% (10 versus 34), the time to progression was 4.0 months (95% CI, 2.856–5.144 months), and the median survival was 12.5 months (95% CI, 11.400–13.600 months) (Figure 1).

### 3.2. RRM1, ERCC1, and BRCA1 mRNA Expression Levels

Tumor RRM1, ERCC1, and BRCA1 mRNA expression levels were analyzed by quantitative RT-PCR. Median mRNA expression levels were 0.2985 (0.001305–2.0281) for RRM1, 0.2811 (0.0144–1.3298) for ERCC1, and 0.3537 (0.0054–38.8020) for BRCA1.

Peripheral blood RRM1, ERCC1, and BRCA1 mRNA expression levels were also measured. Median mRNA expression levels were 0.0115 (0.0004–0.5743) for RRM1, 0.1210 (0.0011–0.8716) for ERCC1, and 0.0632 (0.0004–24.1656) for BRCA1.

**Figure 2:**
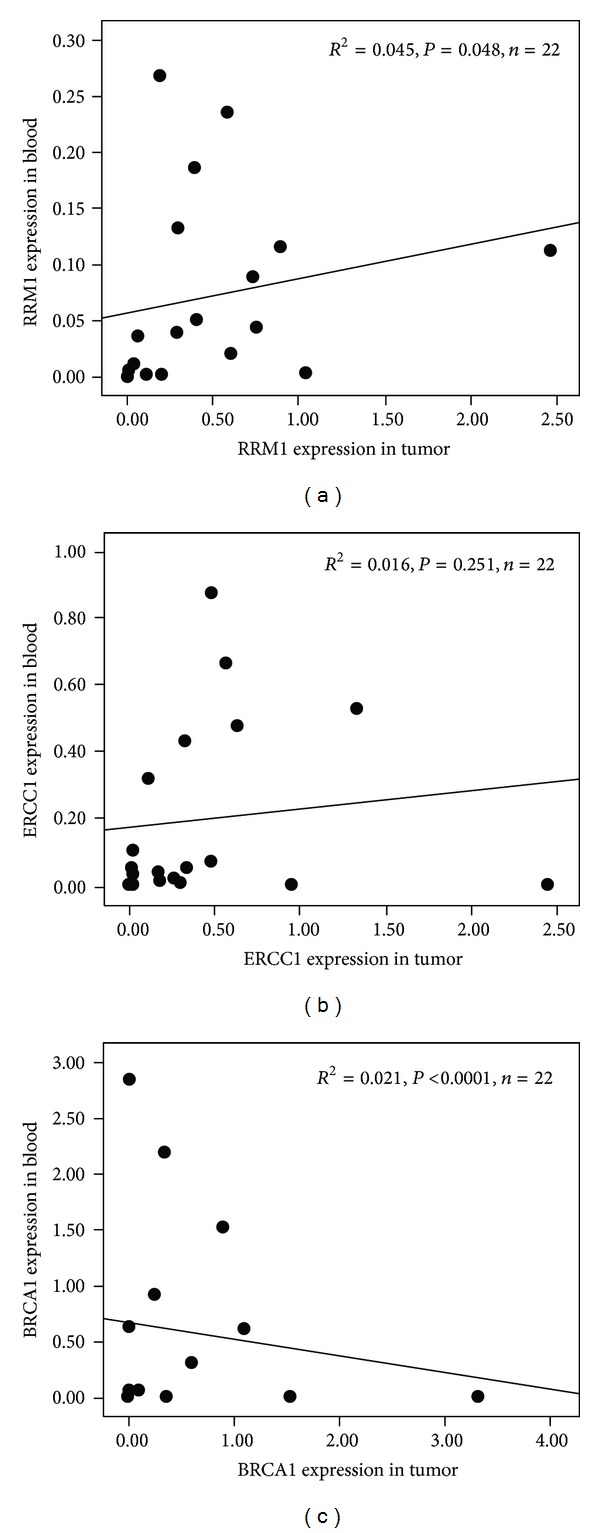
Correlation between RRM1 (a), ERCC1 (b), and BRCA1 (c) mRNA levels in peripheral blood and tumor tissue.

Expression levels in primary tumor tissue and peripheral blood were evaluated for 22 patients. The results indicated that RRM1 expression levels between peripheral blood and tumor tissue were linearly correlated (*R* ^2^ = 0.045, *P* = 0.048) and similar linear correlations were observed for BRCA1 expression (*R* ^2^ = 0.021, *P* < 0.001). This relationship was not observed for ERCC1 expression (*R* ^2^ = 0.016, *P* = 0.251; Figure 2).

### 3.3. Relevance of Peripheral Blood RRM1, ERCC1, and BRCA1 mRNA Expression in Advanced-Stage NSCLC

**Figure 3:**
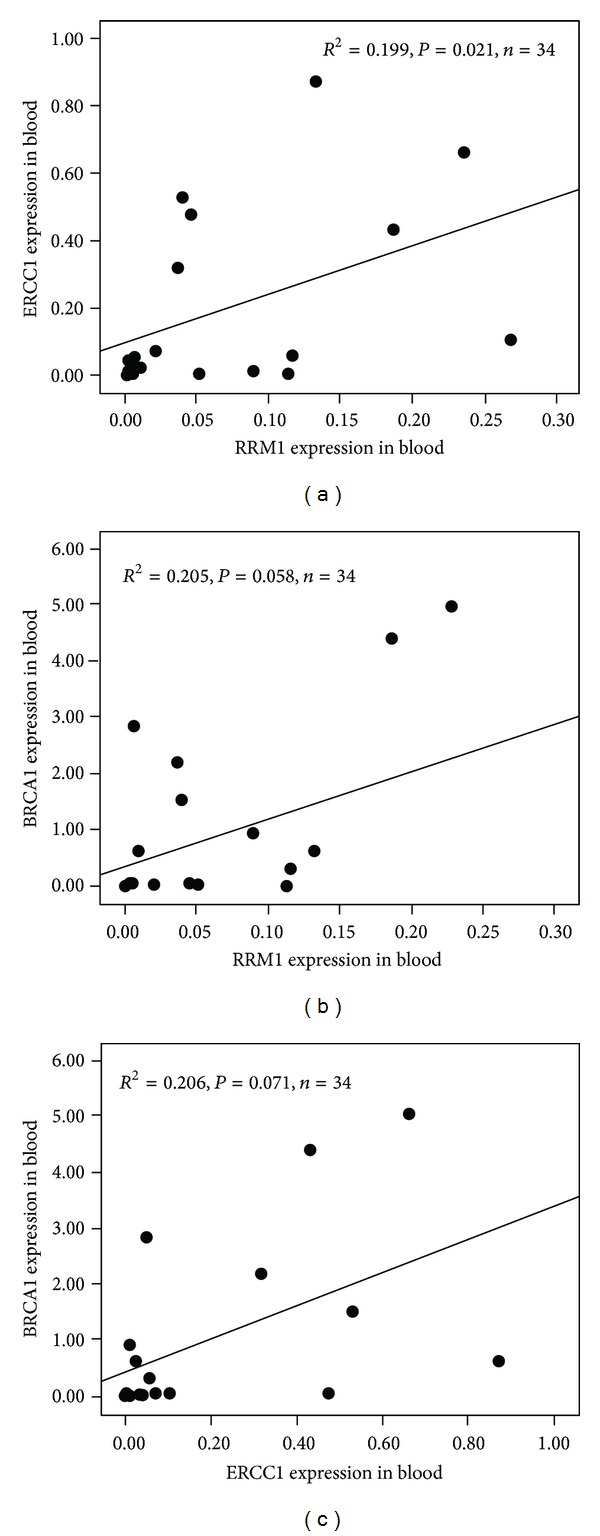
Correlation between RRM1 and ERCC1 (a), RRM1 and BRCA1 (b), and ERCC1 and BRCA1 (c) mRNA levels in peripheral blood.

Real-time PCR was used to detect RRM1, ERCC1, and BRCA1 mRNA expression levels in the peripheral blood of 34 patients with advanced-stage NSCLC. The results indicated a linear correlation (*R* ^2^ = 0.199,*P* = 0.021) between RRM1 and ERCC1 and no linear correlation (Figures 3(a), 3(b), and 3(c)) between RRM1 and BRCA1 (*R* ^2^ = 0.205, *P* = 0.058) and between ERCC1 and BRCA1(*R* ^2^ = 0.206,*P* = 0.071).

### 3.4. The Relationship between Clinical Features and RRM1, ERCC1, or BRCA1 Expression

In 34 patients with advanced NSCLC, the overall response rate was defined as confirmed complete and partial responses and then was the response rate calculated. We investigated the relationship between peripheral blood RRM1, ERCC1, and BRCA1 expression levels with histopathology classifications (squamous cell carcinoma, adenocarcinoma), smoking, age, clinical staging (IIIB and IV), and chemotherapy response. Gene expression (normalized to *β*-actin expression) was categorized into high-expression and low-expression groups where the boundary was defined by the median (Table 2). The results demonstrated no significant differences in RRM1, ERCC1, and BRCA1 expression between/among different clinical stages or smoking statuses or histopathologic classifications (*P* > 0.05). In terms of the tumor susceptibility to chemotherapy, the response rate in the low-RRM1-expression group was significantly greater than in the high-expression group (52.9% versus 5.9%, *χ* ^2^ test, *P* = 0.007). However, ERCC1 and BRCA1 expression between the two groups was not statistically significant (29.4% versus 29.4%, *χ* ^2^ test, *P* = 1.000, and 41.2% versus 17.6%, *χ* ^2^ test, *P* = 0.259).

### 3.5. The Relationship between Survival and RRM1, ERCC1, or BRCA1 Expression

In the univariate analysis model of survival, RRM1, ERCC1, and BRCA1 expression levels in peripheral blood (normalized to *β*-actin expression) of 34 NSCLC cases were divided into low-expression and high-expression groups, where the boundary was defined by the median (median RRM1, ERCC1, and BRCA1 expressions were used as the cutoff values). The median survival of the low-RRM1-expression group was significantly longer than that of the high-expression group (15.5 versus 12.0 months; logrank 3.980, *P* = 0.046). However, no significant differences (*P* > 0.05) in survival were observed between the low-expression and high-expression groups in ERCC1 (15.5 versus 13.0 months; logrank 1.124) or BRCA1 (13.0 versus 15.0 months; logrank 0.702) (Table 3). No significant correlation was noted between survival and age, smoking status, and various histopathologic classifications (squamous cell carcinoma, adenocarcinoma) or clinical stages (IIIB and IV) (*P* > 0.05).

Multivariate analysis using Cox-regression analysis showed that, for patients with NSCLC treated with first-line gemcitabine and carboplatin combination chemotherapy, RRM1 gene expression in peripheral blood may be a predictor of survival and tumor susceptibility to chemotherapy (HR 2.574, 95% CI 0.886–7.479, *P* = 0.082; Table 3).

**Figure 4:**
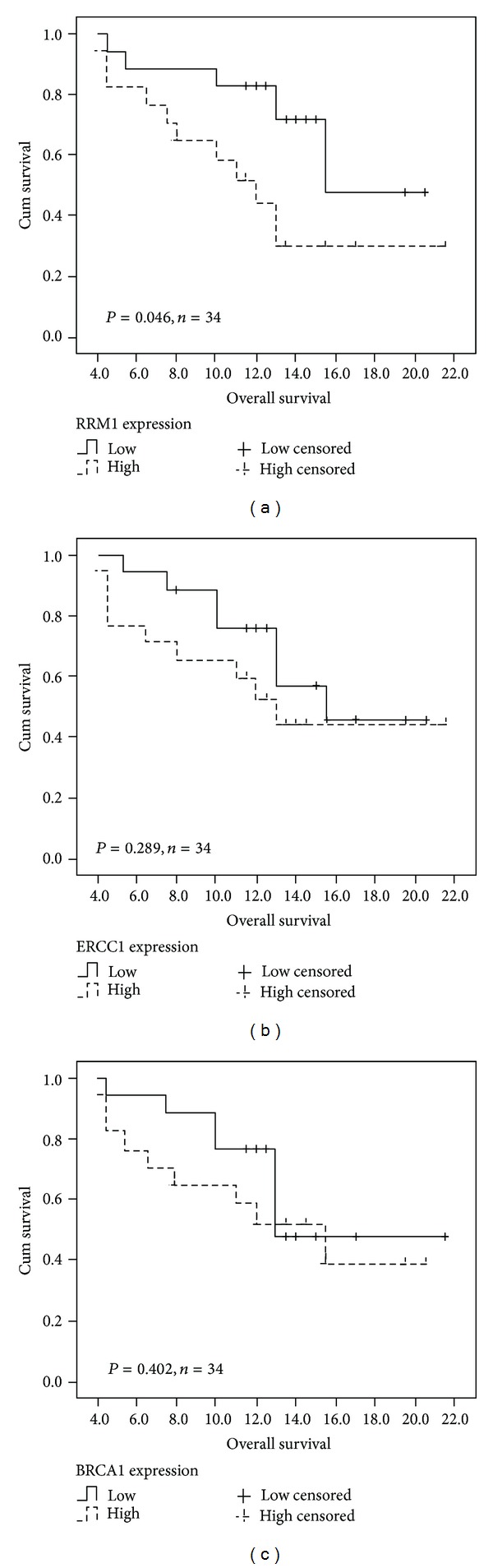
Kaplan-Meier survival analyses for RRM1 (a), ERCC1 (b), and BRCA1 (c) in Chinese patients with NSCLC based on median values of gene expression in overall population.

Kaplan-Meier survival analyses for RRM1, ERCC1, and BRCA1 in subjects with NSCLC based on median gene expression levels in the overall population are shown in Figure 4.

## 4. Discussion

Lung cancer is one of the malignant tumors with the global incidence and case mortality rate. About 85% of lung cancer diagnoses are NSCLC. The majority of patients have lost the opportunity of surgical resection due to the advanced stage at diagnosis, and chemotherapy and radiotherapy are the major treatments for these patients. The use of the third-generation chemotherapy drugs gemcitabine and carboplatin in combination as first-line chemotherapy can extend survival and improve the quality of life for patients with NSCLC [9, 10]. However, clinical studies have found a large disparity in the efficacy of gemcitabine chemotherapy, where only a small number of patients benefit. The efficiency rate is only 20% to 40%, and the median length of survival is 8 to 10 months, and the five-year survival rate is less than 15%. For a fair number of patients, gemcitabine is ineffective but causes toxic side effects. Therefore, the detection of genes, proteins, and other biologic indicators may facilitate the selection of individuals or groups that can benefit the most from chemotherapy. The screening of patients may help achieve individualized therapy for NSCLC and improve individual outcomes and lower toxicity and side effects. Altogether, this research has become an area of intense study [11, 12].

Nucleotide excision repair (NER) that plays important roles in the DNA damage repairing is believed to be involved in the repair of DNA damage induced by chemotherapy. RRM1 and ERCCl are two important members of the NER family. RRM1 is the M1 regulatory subunit of ribonucleotide reductase. It is the nucleotide-binding site, and it controls substrate specificity and the enzyme activity. It is also the binding site for gemcitabine and other nucleoside analogues. The RRM1 gene may also be an important site involved in gemcitabine resistance. In advanced-stage NSCLC patients receiving gemcitabine and carboplatin combination chemotherapy, RRM1 mRNA expression levels affect survival, suggesting RRM1 as an important predictor of gemcitabine resistance [13, 14]. In recent years, increased attention was to the relationship between ERCC1 gene expression level and carboplatin-based chemotherapy sensitivity. A negative correlation has been observed between ERCC1 mRNA expression level and carboplatin drug efficacy. Another important gene in cancer, BRCA1, is involved in mitosis and also plays a key role in the repair of DNA damage. Research indicated that lowering the level of BRCA1 mRNA could increase carboplatin sensitivity in breast cancer cell lines and lead to antimicrotubule drug resistance [15].

However, tumor detection can only be performed in patients who can provide sufficient amounts of tissue sample through surgery or bronchofiberscopy, while many patients with advanced-stage NSCLC who have a cytopathologically confirmed diagnosis do not possess sufficient quantities of tissue sample and only a small number of cancer cells are shed into the peripheral blood. Hence, these patients are unable to undergo such tests. Presently, the detection of gene expression in peripheral blood is convenient and widely used. Therefore, we can take advantage of using peripheral blood for the detection of gene expression to predict tumor cell response to gemcitabine and carboplatin combination therapy.

In the present study, 40 patients with histologically confirmed stages IIIB and IV NSCLC were recruited. Genetic testing of tumor tissue and peripheral blood was performed in 22 cases. Through correlation analysis of RRM1, ERCC1, and BRCA1 mRNA expression levels, our results indicated a linear correlation between peripheral blood and tumor tissue RRM1 expression. And a linear correlation was also observed for BRCA1. However, no linear correlation was detected for ERCC1.

The predictive and prognostic values were analyzed for peripheral blood RRM1, ERCC1, and BRCA1 mRNA expression in 34 patients. Gene expression (normalized to *β*-actin) was classified into high-expression and low-expression groups, where the boundary was defined by the median. The results indicated no significant differences in RRM1, ERCC1, and BRCA1 expression levels among age, smoking status, and various pathologic classifications (squamous cell carcinoma, adenocarcinoma) or clinical stages (IIIB and IV). The response rate of the low-RRM1-expression group was higher than that of the high-expression group (52.9% versus 5.9%, *χ* ^2^ test, *P* = 0.007), while no differences were detected for ERCC1 or BRCA1 expression (*P* = 1.000 and *P* = 0.259).

Factors affecting late-stage NSCLC patient survival were analyzed with the COX proportional hazards model. The median survival of the low-RRM1-expression group was significantly longer than that of the high-expression group (15.5 versus 12.0 months; logrank 3.980, *P* = 0.046), while no significant correlations between gene expression and survival were detected in both the high and low ERCC1 and BRCA1 groups. In addition, age, smoking, histopathologic classifications (squamous cell carcinoma, adenocarcinoma), and clinical staging (IIIB and IV) were not associated with survival. This evidence suggests that low RRM1 expression can increase the chemotherapeutic efficacy and survival in NSCLC patients. Our study also confirmed previously suggested evidence of the relationship between tumor tissue RRM1 expression levels and gemcitabine and carboplatin combination chemotherapy prognosis, which can be demonstrated through peripheral blood RRM1 levels.

In conclusion, this study indicated that low peripheral blood RRM1 was correlated with high chemotherapy efficacy and increased survival in NSCLC patients receiving gemcitabine and carboplatin combination therapy. Peripheral RRM1 gene expression may be a predictor of chemotherapy sensitivity and overall survival (HR 2.574, 95% CI 0.886–7.479, *P* = 0.082). These results will assist in the selection of patients most suitable for receiving gemcitabine and carboplatin combination chemotherapy.

## Figures and Tables

**Table 1:** Baseline patients characteristics (*n* = 34).

Characteristics	Frequency	Percentage (%)
Age (years)		
Median	61	
Range	40–80	
Sex		
Male	25	74
Female	9	26
Smoker		
Yes	13	38
No	21	62
Performance status		
0	9	26
1	24	71
2	1	3
Stage		
IIIB	9	26
IV	25	74
Histology		
Squamous cell carcinoma	10	29
Adenocarcinoma	24	71
Metastasis sites		
Lung	7	21
Liver	3	9
Bone	13	38
Nodes	8	24
Others	8	24
Number of metastasis sites		
1	10	29
2	8	24
≥3	4	12

**Table 2:** Expression of RRM1, ERCC1, and BRCA1 in the group of different histological types, TMN stages, and response to chemotherapy.

Characteristic	RRM1			ERCC1			BRCA1		
	Low	High	*P*	Low	High	*P*	Low	High	*P*
Age									
>65	5	6	1.000	6	5	1.000	6	5	1.000
<65	12	11		11	12		11	12	
Smoker									
Yes	7	6	1.000	8	5	0.290	8	5	0.290
No	10	11		9	12		9	12	
Stage									
IIIB	4	5	1.000	5	4	1.000	5	4	1.000
IV	13	12		13	12		12	13	
Histology									
Squamous cell carcinoma	5	5	1.000	3	7	0.132	4	6	0.708
Adenocarcinoma	12	12		14	10		13	11	
Response									
Partial	9	1	0.007	5	5	1.000	7	3	0.259
Stable/progressive	8	16		12	12		10	14	

**Table 3:** Factors associated with overall survival time of Chinese subjects with NSCLC.

Factors and gene expression	Number of patients	Median survival time (month)	Univariate analysis		Multivariate analysis		
			Logrank	*P*	HR	95% CI	*P*
Age							
>65	11	12.0	0.065	0.799	—	—	—
<65	23	12.5			—	—	—
Smoker							
Yes	13	12.0	0.008	0.927	—	—	—
No	21	13.0			—	—	—
Stage							
IIIB	9	12.0	0.169	0.681	—	—	—
IV	25	13.0			—	—	—
Histology							
Squamous cell carcinoma	10	11.0	2.801	0.094	—	—	—
Adenocarcinoma	24	13.0			—	—	—
Response							
Partial	10	12.5	16.154	0.0001	0.614	0.267–2.468	0.017
Stable/progressive	24	12.0			1		
RRM1							
Low	17	15.5	3.980	0.046	2.574	0.886–7.479	0.082
High	17	12.0			1		
ERCC1							
Low	17	15.5	1.124	0.289	—	—	—
High	17	13.0			—	—	—
BRCA1							
Low	17	13.0	0.702	0.402	—	—	—
High	17	15.5			—	—	—

## Abbreviations

RRM1: Ribonucleotide reductase M1
ERCCl: Excision repair cross complementation 1
BRCA1: Breast cancer susceptibility gene 1
NSCLC: Nonsmall cell lung cancer.
